# Consumption of Dietary Fiber in Relation to Psychological Disorders in Adults

**DOI:** 10.3389/fpsyt.2021.587468

**Published:** 2021-06-24

**Authors:** Faezeh Saghafian, Nafiseh Sharif, Parvane Saneei, Ammar Hassanzadeh Keshteli, Mohammad Javad Hosseinzadeh-Attar, Hamid Afshar, Ahmad Esmaillzadeh, Peyman Adibi

**Affiliations:** ^1^Department of Community Nutrition, School of Nutritional Sciences and Dietetics, Tehran, University of Medical Sciences, Tehran, Iran; ^2^Food Security Research Center, Department of Community Nutrition, School of Nutrition and Food Science, Isfahan University of Medical Sciences, Isfahan, Iran; ^3^Department of Medicine, University of Alberta, Edmonton, AB, Canada; ^4^Isfahan Gastroenterology and Hepatology Research Center, Isfahan University of Medical Sciences, Isfahan, Iran; ^5^Department of Clinical Nutrition, School of Nutritional Sciences and Dietetics, Tehran, University of Medical Sciences, Tehran, Iran; ^6^Psychosomatic Research Center, Department of Psychiatry, Isfahan University of Medical Sciences, Isfahan, Iran; ^7^Obesity and Eating Habits Research Center, Endocrinology and Metabolism Molecular-Cellular Sciences Institute, Tehran University of Medical Sciences, Tehran, Iran; ^8^Department of Community Nutrition, School of Nutrition and Food Science, Isfahan University of Medical Sciences, Isfahan, Iran

**Keywords:** fiber, depression, anxiety, distress, nutritional epidemiology

## Abstract

**Background:** Previous observational studies suggested a relationship between dietary fiber consumption and mental health, but the findings were conflicting. We evaluated the link between dietary fiber intake and prevalence of depression, anxiety, and psychological distress among a large population of Iranian adults.

**Methods:** A cross-sectional study among 3,362 Iranian adults working in 50 health centers was done. Data of dietary intakes were collected through a validated semiquantitative dish-based 106-item food frequency questionnaire (FFQ). Anxiety, depression, and psychological distress were defined based on the Iranian validated version of Hospital Anxiety and Depression Scale (HADS) and General Health Questionnaire (GHQ).

**Results:** After adjustment for potential confounders, participants in the top quartile of total dietary fiber intake had a 33% and 29% lower risk of anxiety and high psychological distress [odds ratio (OR): 0.67; 95% CI: 0.48, 0.95 and OR: 0.71; 95% CI: 0.53, 0.94, respectively] compared to the bottom quartile of intake. The highest total dietary fiber intake was also inversely related to a lower risk of depression in women (OR: 0.63; 95% CI: 0.45, 0.88) but not in men. Among overweight or obese participants, higher intake of dietary fiber was related to a decreased risk of high psychological distress (OR: 0.52; 95% CI: 0.34, 0.79). A high level of dietary fiber intake was related to a lower risk of anxiety in normal-weight individuals (OR: 0.50; 95% CI: 0.31, 0.80).

**Conclusion:** Significant inverse associations between total dietary fiber intake with anxiety and high psychological distress were found in Iranian adults. More consumption of dietary fiber was also related to reduced odds of depression in women. More investigations with prospective nature are needed to affirm these findings.

## Introduction

The prevalence of psychological disorders, including depressive symptoms and anxiety, is increasing worldwide. Depression as a highly prevalent mental disorder is a major public health problem in the general population (1). The World Health Organization (WHO) has reported that 4.4% of the world's population suffers from depression (1). Also, the total number of people with depression was estimated to exceed 300 million in 2015 (1). Based on the Global Burden of Disease Study, the total estimated number of people living with depression increased by 18.4% between 2005 and 2015 (2). Depression is a leading cause of disability worldwide, and it could additionally increase the risk of coronary disease (2). Anxiety, another mental disorder, has recently attracted a lot of attention due to its high prevalence and relationship with chronic diseases (3). In the United States, the prevalence of depression is 19.2%, and 1.7 times more likely to occur among women than men (4). The prevalence of anxiety and depression was respectively 21.0 and 20.8% in Iranian adults (5). Mental disorders reduced productivity, lowered quality of life, and increased the rate of disabilities, morbidity, and fatality, along with a significant increase in cost to both individuals and society (6, 7). Finding related risk factors to these mental disorders is a high priority in public health (8).

Several studies have focused on diet as a modifiable risk factor that might affect the development of mental disorders. Prior researches investigated the relationship between intake of specific nutrients or foods and psychological disorders (9). Dietary patterns, such as Mediterranean diet, are also related to depression (10). The results of two systematic reviews (11, 12) and one meta-analysis (13) suggested that a healthy dietary pattern, consisting of high consumption of fruit, vegetables, and whole grains, was associated with decreased odds of depression (13). Other meta-analyses showed a reverse association between fruit and vegetable consumption with anxiety and depressive symptoms (14, 15). Fruits and vegetables are rich sources of bioactive compounds such as vitamins, antioxidants, and dietary fiber that have been shown to be related to a reduced depression risk (16).

Although the association of dietary fiber and mental disorders was previously studied (6), the results were inconsistent (17). In Hong Kong, a cross-sectional study has shown a reverse association between fiber intake and depression in elders (18). Another investigation showed that more consumption of dietary fiber from fruits and vegetables decreased the risk of depressive symptoms, although no significant relation was found in case of total, soluble, insoluble fiber, or fiber from cereals (6). Oishi et al. (9) have also reported a non-significant association between fiber intake and depression in old adults. To the best of our knowledge, no previous study has investigated the linkage between dietary fiber intake and mental disorders among Middle Eastern populations and there was no investigation with regard to the relation to anxiety in the world. Different dietary intakes and lifestyle in the Middle East in comparison with Western countries might affect the development of mental disorders. Therefore, we evaluated the relationship between dietary fiber and mental disorders (depression, anxiety, and psychological distress) in Iranian adults. We hypothesized that more consumption of total dietary fiber would be related to decreased odds of psychological disorders.

## Materials and Methods

### Participants

This study was done within the SEPAHAN (Study on the Epidemiology of Psychological-Alimentary Health and Nutrition) project (19), a cross-sectional investigation that studied Iranian general adults working in several healthcare centers (*n* = 50) affiliated to Isfahan University of Medical Sciences (IUMS). The information was obtained in two steps. First, data of sociodemographic variables and dietary intakes of 10,087 individuals with the age of 18–55 years were collected through a detailed self-administered questionnaire; 8,691 participants replied to the questionnaire (response rate: 86.2%). Then, data of psychological disorders were gathered 1 month later through standardized questionnaires (response rate: 61.8%). No significant difference was found in demographic characteristics of participants who completed and returned the questionnaires with non-respondents. Acquired information from these two steps was integrated. In the present study, four groups of respondents were discarded: (1) those who did not return the questionnaires in one of the steps; (2) those who did not report their identification number in step 1 or 2; (3) those with incomplete information with regard to the exposure, outcome, or covariate variables; (4) those receiving energy intakes of <800 kcal/day or more than 4,200 kcal/day (20). Since energy intakes outside the range of 800–4,200 kcal/day were unlikely to be true for even relatively inactive women and active men, we considered these subjects as underreporters and overreporters of energy intake and excluded them from the analysis. Finally, the information of dietary intakes and psychological disorders was gathered for 3,362 individuals. An informed written consent was signed by each participant, and the Medical Research Ethics Committee of Tehran University of Medical Sciences (TUMS), Tehran, Iran, has ethically approved this study (no. IR.TUMS.VCR.REC.1395.289).

### Assessment of Total Dietary Fiber Intake

Dietary intakes were determined by the use of a validated 106-item dish-based semiquantitative food frequency questionnaire (FFQ). This Willett-format FFQ was previously designed for the Iranian adult population (21). More information with regard to the design, various included foods, and validity of the applied FFQ was previously published (19). Briefly, information on the frequency of raw foods or cooked foods as mixed dishes consumption over the past year with common portion sizes among Iranians was included in this FFQ. Because dietary intakes tend to be reasonably correlated from year to year, we have asked the participants to describe their frequency of using foods in reference to the preceding year, although some sort of recall bias might occur (20). Nutrient intakes, including total dietary fiber, were calculated by summing up the nutrient contents of all included foods or dishes. For each participant, nutrient intakes were obtained by the use of a modified version of Nutritionist IV software based on Iranian foods. Based on our previous investigations, the applied FFQ had reasonable validity and reliability and could reasonably categorize usual intake of special foods (22), food groups (23), or nutrients (24) in Iranian adults.

### Assessment of Psychological Disorders

To define depression and anxiety, the validated Iranian version of Hospital Anxiety and Depression Scale (HADS) was applied (25). It is a brief and practical questionnaire that screens the symptoms of depression and anxiety. It has two subscales of anxiety and depression with 14 questions. Each item or question had a 4-point scale, “scored 0–3.” So, each subject could obtain a maximum score of 21 for anxiety or depression; higher scores, higher level of anxiety or depression. In the current study, obtaining a score of 0–7 was defined as being normal or having mental health, while scores of 8 or more on the subscale of anxiety or depression were considered being anxious or depressed. Based on a validation study of HADS among Iranian adults, Cronbach's alpha coefficients were 0.78 for anxiety subscale and 0.86 for depression subscale (25). A validated Iranian version of 12-item General Health Questionnaire (GHQ) was used to assess psychological distress (Cronbach's alpha coefficient = 0.87) (26). It is a brief and useful tool to screen distress levels. The respondents are asked if they have recently experienced a particular psychological distress symptom or not. Each question contains a 4-point scale, “scored 0–3.” Two common methods of bimodal (0-0-1-1) or Likert (0-1-2-3) could be used for scoring. Since the bimodal fashion of scoring appeared to be useful and efficient in Iranian adults (26), the bimodal style was used for scoring in this study. This method provided a score from 0 to 12 for each participant; higher scores, higher level of psychological distress. A score of 4 or more was considered having a high level of psychological distress in the current study (26).

### Assessment of Confounding Variables

Pretested self-administered questionnaires were used to gather information on age, sex, diabetes, education, marital status, household size, home ownership, smoking status, use of antidepressant medication (including fluoxetine, fluvoxamine, nortriptyline, citalopram, amitriptyline or imipramine, and sertraline), and dietary supplement use (including vitamins, iron, calcium, and other dietary supplements) of the participants. In addition, we used a validated self-reported questionnaire to obtain information of height (cm) and weight (kg) (27). Body mass index (BMI) was calculated as body weight (kg) divided by the square of height (m^2^). Subjects were classified based on their BMI into normal-weight group (<25 kg/m^2^) and overweight or obese group (≥25 kg/m^2^). Using a General Practice Physical Activity Questionnaire (GPPAQ), physical activity of study participants was also assessed. This questionnaire is a simple validated screening tool that ranks individuals based on their physical activity into four groups by focusing on their current general activities.

### Statistical Methods

First, energy-adjusted total dietary fiber intake of the study subjects was obtained based on residual method by the use of the regression model with total caloric intake as the independent variable and absolute fiber intake as the dependent variable (20). Then, participants were classified into quartiles of energy-adjusted dietary fiber intakes. The model assumption was checked before the analysis. All assumptions such as normal distribution of independent variables and homogeneity of variances were met due to the large study sample size. Continuous variables across different categories of dietary fiber intakes were compared by the use of one-way ANOVA. The *post-hoc* test of Bonferroni was also administered to correct multiple testing. Categorical variables across different categories of dietary fiber intakes were compared through chi-square test. Analysis of covariance (ANCOVA) was used to compare the intakes of several nutrients and food groups, while age, gender, and energy intake were adjusted. Logistic regression in different models was used to assess the relationships between dietary fiber intakes with depression, anxiety, and psychological distress. The covariates were selected based on previous investigations (6, 7, 28). First, the relationships were examined in a crude model. Then, adjustments were done for age (years), sex (male vs. female), and energy intake (kcal/day). We further controlled for smoking (current smokers vs. ex-smokers or non-smokers), physical activity (≥1 vs. <1 h/week), marital status (single/divorced/widow vs. married), self-reported diabetes (yes vs. no), socioeconomic status (SES) [including educational level (>diploma vs. ≤ diploma), household size (>4 vs. ≤ 4 members), house possession (yes vs. no)], antidepressant medication use (yes vs. no), and dietary supplement use (yes vs. no) in the second model. In order to find independent associations from dietary intakes, in the third model, dietary intakes that might be related to psychological disorders (6, 7, 28) [including fat intake, n-3 fatty acids, vitamin B group (B_1_, B_2_, B_6_, folate, pantothenic acid), and total antioxidants (vitamins C, E, selenium, beta carotene)] were also considered. In the last model, more adjustment for BMI was done. To obtain the trend of odds ratios (ORs) across categories of dietary fiber intakes, quartiles of intake were considered as an ordinal variable. In all models, subjects in the first category of energy-adjusted total dietary fiber intake were considered the reference category. Significant interactions between sex and BMI groups with prevalence of psychological disorders emerged (*p* < 0.05). So, stratified analyses by sex and BMI groups were applied. Statistical Package for the Social Sciences (SPSS Inc., version 18.0, Chicago, IL) software was used for the analyses. *p*-values < 0.05 were considered statistically significant.

## Results

The study subjects consisted of 3,362 individuals with a mean age of 36.3 years and mean weight of 68.7 kg; 58.3% of participants were females. Depression, anxiety, and high psychological distress were respectively prevalent among 30.0, 15.2, and 25.0% of participants [among males: 22.9, 10.8, 19.7%; among females: 35.1, 18.3, 28.7%, respectively]. General characteristics of study subjects across quartiles of energy-adjusted dietary fiber intake are provided in Table 1. In comparison to those in the bottom quartile, individuals in the top quartile of dietary fiber intake were more likely to be female, older, physically active, and overweight or obese. Other sociodemographic characteristics of participants across different quartiles of fiber intake were not significantly different.

**Table 1:** General characteristics of study participants across quartiles of energy-adjusted dietary fiber intake (*n* = 3,362)^a^.

	**Quartiles of energy-adjusted dietary fiber intake**				
	**(*n* = 840) (<19 g/day)**	**(*n* = 841)(19–22.1 g/day)**	**(*n* = 841) (22.2–25.6 g/day)**	**(*n* = 841)(>25.6 g/day)**	***p*^**b**^**
Age (years)	35.50 ± 7.66	35.95 ± 8.09	36.25 ± 7.55	37.43 ± 8.05	<0.001
Weight (kg)	69.22 ± 13.63	69.14 ± 13.68	67.61 ± 12.57	68.64 ± 12.78	0.05
BMI (kg/m^2^)	24.70 ± 3.81	24.86 ± 3.89	24.80 ± 3.78	25.25 ± 3.79	0.24
Female (%)	51.7	55.3	61.1	65.0	<0.001
Marital status (%)					0.69
Married	81.5	82.3	80.4	82.6	
Single	16.8	16.2	17.3	16.2	
Divorced/widow	1.7	1.5	2.3	1.2	
Education (%)					0.84
Under diploma	10.6	11.7	13.0	12.9	
Diploma	27.5	27.8	26.5	26.0	
Above diploma and under master's	53.4	53.5	53.5	53.5	
Master's and above	8.5	7.1	6.9	7.6	
Household size (%)					0.02
<3	42.7	40.4	36.3	36.3	
3–4	46.2	47.4	49.1	50.8	
>4	11.1	12.2	14.6	12.9	
House possession (%)	56.4	58.7	57.9	60.0	0.06
Diabetes (%)	1.8	1.5	1.8	2.0	0.90
Antidepressant medications^c^ (%)	4.5	5.7	5.7	6.3	0.44
Dietary supplement use^d^ (%)	28.9	28.1	31.2	31.9	0.27
Smokers (%)	15.5	14.1	12.1	13.5	0.25
Physically activity (%)					0.004
Never	42.3	42.0	39.0	34.4	
<1 h/week	24.9	27.8	28.8	26.4	
1–3 h/week	22.5	19.4	20.9	24.0	
>3 h/week	10.2	10.8	11.3	15.2	
Obese^e^ (%)	41.8	43.9	44.7	48.8	0.03

a *All values are means ± standard deviation (SD), unless indicated*.

b *Obtained from ANOVA for continuous variables and chi-square test for categorical variables*.

c *Antidepressant medications include nortriptyline, amitriptyline or imipramine, fluoxetine, citalopram, fluvoxamine, and sertraline*.

d *Dietary supplements include iron, calcium, vitamins, and other dietary supplements*.

e *Body mass index (BMI) ≥25 kg/m^2^*.

Dietary intakes of different nutrients and food groups across quartiles of energy-adjusted fiber intake are provided in Table 2. Participants in the highest category of fiber intake had significantly higher intakes of proteins, carbohydrates, vitamin B_1_, vitamin B_6_, iron, vitamin C, whole grains, fruit, vegetables, nuts, soy, and legumes in comparison to those in the lowest category, while subjects in the bottom quartile of fiber intakes, in comparison to those in the top quartile, had more intakes of energy, fats and red meat, refined grains, dairy products, omega-3 fatty acids, and vitamin E.

**Table 2:** Dietary intakes of selected nutrients and food groups across quartiles of energy-adjusted dietary fiber intake (*n* = 3,362)^a^.

	**Quartiles of energy-adjusted dietary fiber intake**				
	**(*n* = 840) (<19 g/day)**	**(*n* = 841)(19–22.1 g/day)**	**(*n* = 841) (22.2–25.6 g/day)**	**(*n* = 840)(>25.6 g/day)**	***p*^**b**^**
Energy (kcal/day)	2,530.60 ± 29.70	2,221.13 ± 29.74	2,261.33 ± 29.35	2,513.06 ± 29.27	<0.001
**Nutrients**					
Proteins (% of energy)	14.67 ± 0.08	15.00 ± 0.08	14.88 ± 0.08	14.76 ± 0.08	0.03
Fats (% of energy)	40.39 ± 0.23	38.30 ± 0.23	36.87 ± 0.23	34.55 ± 0.22	<0.001
Carbohydrates (% of energy)	45.72 ± 0.27	47.84 ± 0.27	49.85 ± 0.26	53.04 ± 0.26	<0.001
Dietary fiber (g/day)	15.90 ± 0.10	20.69 ± 0.10	23.86 ± 0.10	29.75 ± 0.10	<0.001
Omega-3 fatty acids (g/day)	1.78 ± 0.03	1.78 ± 0.03	1.78 ± 0.03	1.64 ± 0.03	<0.001
Vitamin B1 (mg/day)	1.62 ± 0.02	1.78 ± 0.02	1.94 ± 0.02	1.96 ± 0.02	<0.001
Vitamin B6 (mg/day)	1.82 ± 0.01	1.94 ± 0.01	2.00 ± 0.01	2.17 ± 0.01	<0.001
Iron (mg/d)	16.08 ± 0.11	17.71 ± 0.11	18.19 ± 0.11	18.46 ± 0.11	<0.001
Vitamin C (mg/day)	74.03 ± 1.70	89.48 ± 1.71	105.27 ± 1.68	136.82 ± 1.68	<0.001
Vitamin E (mg/day)	22.22 ± 0.22	21.64 ± 0.22	21.04 ± 0.21	20.91 ± 0.21	<0.001
**Food groups (g/day)**					
Red meat	78.31 ± 1.49	80.62 ± 1.49	78.60 ± 1.47	76.81 ± 1.46	<0.001
Whole grains	14.02 ± 2.63	32.61 ± 2.64	39.80 ± 2.60	82.50 ± 2.60	<0.001
Refined grains	438.46 ± 5.88	411.92 ± 5.90	394.29 ± 5.81	329.09 ± 5.79	<0.001
Fruit	193.24 ± 7.44	257.75 ± 7.45	337.33 ± 7.34	476.80 ± 7.32	<0.001
Vegetables	198.11 ± 4.15	224.14 ± 4.16	242.84 ± 4.01	290.19 ± 4.09	<0.001
Nuts, soy and legumes	40.78 ± 1.25	52.01 ± 1.26	60.97 ± 1.24	74.55 ± 1.23	<0.001
Dairy	397.58 ± 9.56	352.21 ± 9.58	336.36 ± 9.43	310.51 ± 9.41	<0.001

a *All values are means ± standard error (SE); energy intake is adjusted for age and gender; all other values are adjusted for age, gender, and energy intake*.

b *Obtained from analysis of covariance (ANCOVA)*.

**Figure 1:**
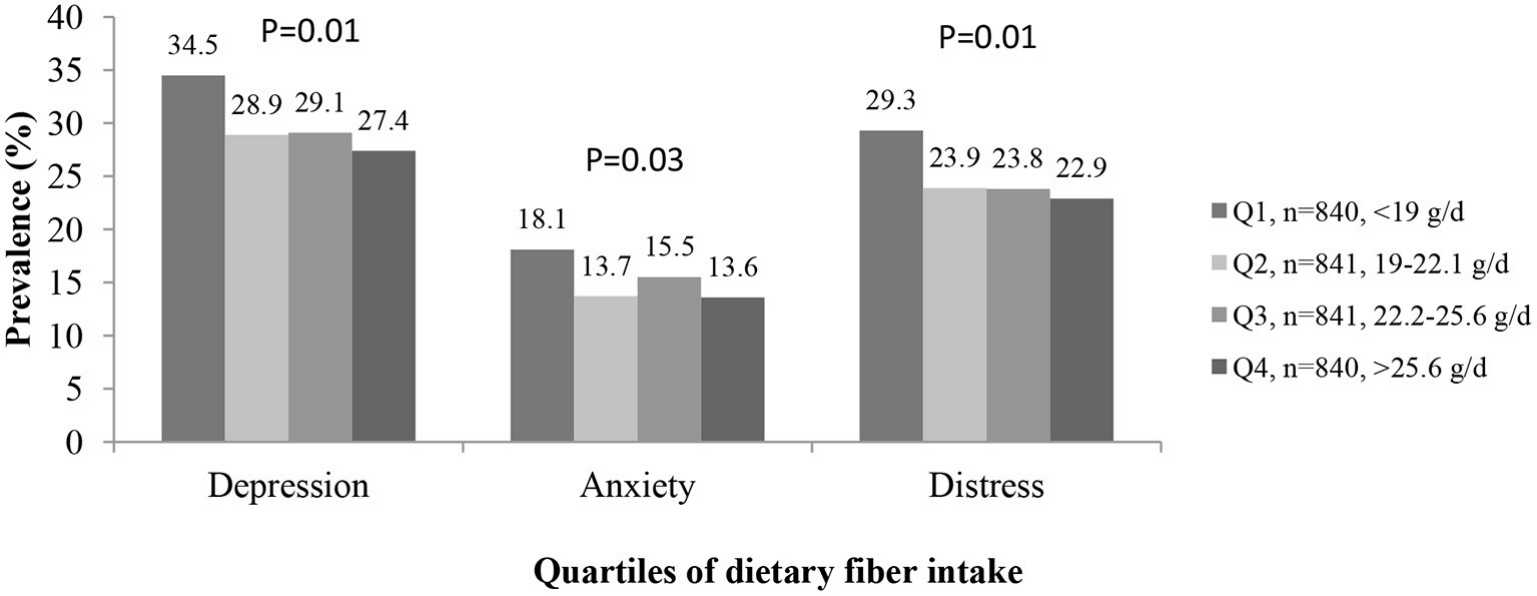
The prevalence of depression, anxiety, and high psychological distress in study participants across different quartiles of energy-adjusted dietary fiber intake (*n* = 3,362).

As shown in Figure 1, participants in the fourth quartile of total dietary fiber intake had lower prevalence of depression (27.4 vs. 34.5 %, *p* = 0.01) and anxiety (13.6 vs. 18.1%, *p* = 0.03) and high psychological distress (22.9 vs. 29.3%, *p* = 0.01) in comparison to those in the first quartile.

Multivariable-adjusted ORs and 95% confidence intervals (CIs) for psychological disorders across different categories of dietary fiber intakes are provided in Table 3. Highest intake of fiber, in comparison to the lowest intake, was significantly associated with lower odds of depression in the crude model (OR: 0.71; 95% CI: 0.58, 0.88). This relationship was significant after adjustment for age, gender, energy intake, antidepressant medications, and dietary supplements, but the association disappeared after adjustments for dietary intakes and BMI (OR: 0.81; 95% CI: 0.62, 1.07).

**Table 3:** Multivariable-adjusted odds ratios and 95% confidence intervals for depression, anxiety, and psychological distress across quartiles of energy-adjusted dietary fiber intake in the whole population (*n* = 3,362)^a^.

	**Quartiles of energy-adjusted dietary fiber intake**				
	**(*n* = 840) (<19 g/day)**	**(*n* = 841)(19–22.1 g/day)**	**(*n* = 841) (22.2–25.6 g/day)**	**(*n* = 840)(>25.6 g/day)**	***p*_**trend**_**
**Depression**					
Crude	1.00	0.77 (0.63–0.95)	0.78 (0.63–0.96)	0.71 (0.58–0.88)	0.003
Model 1	1.00	0.75 (0.60–0.95)	0.75 (0.60–0.94)	0.64 (0.51–0.81)	<0.001
Model 2	1.00	0.75 (0.59–0.94)	0.74 (0.59–0.93)	0.63 (0.50–0.80)	<0.001
Model 3	1.00	0.79 (0.63–1.00)	0.84 (0.66–1.07)	0.79 (0.61–1.03)	0.14
Model 4	1.00	0.81 (0.64–1.03)	0.86 (0.67–1.10)	0.81 (0.62–1.07)	0.20
**Anxiety**					
Crude	1.00	0.72 (0.55–0.93)	0.83 (0.64–1.07)	0.71 (0.55–0.93)	0.03
Model 1	1.00	0.64 (0.48–0.86)	0.77 (0.58–1.00)	0.63 (0.47–0.83)	0.007
Model 2	1.00	0.64 (0.47–0.85)	0.77 (0.58–1.02)	0.61 (0.46–0.82)	0.005
Model 3	1.00	0.64 (0.47–0.86)	0.79 (0.58–1.06)	0.65 (0.46–0.90)	0.04
Model 4	1.00	0.67 (0.49–0.91)	0.87 (0.64–1.18)	0.67 (0.48–0.95)	0.11
**Psychological distress**					
Crude	1.00	0.76 (0.61–0.94)	0.75 (0.61–0.94)	0.71 (0.57–0.89)	0.004
Model 1	1.00	0.77 (0.61–0.98)	0.76 (0.60–0.96)	0.65 (0.51–0.83)	0.001
Model 2	1.00	0.76 (0.60–0.97)	0.75 (0.59–0.95)	0.64 (0.50–0.81)	<0.001
Model 3	1.00	0.77 (0.60–0.98)	0.77 (0.60–1.00)	0.70 (0.53–0.92)	0.02
Model 4	1.00	0.81 (0.63–1.04)	0.85 (0.65–1.10)	0.71 (0.53–0.94)	0.03

a *Model 1: Adjusted for age, gender, and energy intake*.

*Model 2: Further adjustment for physical activity, smoking, marital status, socioeconomic status, diabetes, and use of antidepressant medications and dietary supplements*.

*Model 3: Additional controlling for dietary intakes of fat, n-3 fatty acids, vitamin B group, and total antioxidants*.

*Model 4: Further adjusted for body mass index (BMI)*.

Compared with individuals in the reference category, those in the top category of dietary fiber consumption had significantly lower odds of anxiety both in the crude (OR: 0.71; 95% CI: 0.55, 0.93) and adjusted models, such that after adjustment for all potential confounding variables including BMI, the highest category of dietary fiber intake was associated with a 33% lower odds of anxiety (OR: 0.67; 95% CI: 0.48, 0.95) compared with the lowest intake.

Consumption of dietary fiber was related to lower odds of high psychological distress (OR: 0.71; 95% CI: 0.57, 0.89). Participants in the fourth quartile of dietary fiber intake had 29% lower odds of high psychological distress in comparison to subjects in the first quartile (OR: 0.71; 95% CI: 0.53, 0.94) in the fully adjusted model for potential confounders.

Multivariable-adjusted ORs and 95% CIs for psychological disorders across quartiles of energy-adjusted dietary fiber consumption stratified by gender are provided in Tables 4, 5. Either before or after adjusting for all potential confounding variables, we did not observe any significant association between higher dietary fiber intake and psychological disorder in men. In women, the highest dietary fiber consumption was respectively associated with 41, 35, and 40% lower odds of depression, anxiety, and psychological distress in the crude model. After taking all potential confounding variables into account, this relationship remained significant [for depression (OR: 0.63; 95% CI: 0.45, 0.88), for anxiety (OR: 0.56; 95% CI: 0.37, 0.85), and high psychological distress (OR: 0.70; 95% CI: 0.47, 0.95)].

**Table 4:** Multivariable-adjusted odds ratios and 95% confidence intervals for depression, anxiety, and psychological distress across quartiles of energy-adjusted dietary fiber intake in men (*n* = 1,403)^a^.

	**Quartiles of energy-adjusted dietary fiber intake**				
	**(*n* = 406) (<19 g/day)**	**(*n* = 376)(19–22.1 g/day)**	**(*n* = 327) (22.2–25.6 g/day)**	**(*n* = 294)(>25.6 g/day)**	***p*_**trend**_**
**Depression**					
Crude	1.00	0.93 (0.67–1.28)	0.76 (0.53–1.08)	0.77 (0.54–1.04)	0.08
Model 1	1.00	1.04 (0.72–1.50)	0.78 (0.53–1.16)	0.81 (0.54–1.20)	0.16
Model 2	1.00	1.05 (0.72–1.54)	0.77 (0.52–1.16)	0.85 (0.56–1.27)	0.22
Model 3	1.00	1.23 (0.83–1.81)	0.98 (0.64–1.51)	1.23 (0.77–1.95)	0.61
Model 4	1.00	1.38 (0.92–2.06)	1.08 (0.69–1.69)	1.35 (0.83–2.20)	0.39
**Anxiety**					
Crude	1.00	0.84 (0.54–1.29)	0.70 (0.44–1.11)	0.65 (0.39–1.06)	0.05
Model 1	1.00	0.74 (0.45–1.22)	0.66 (0.39–1.11)	0.67 (0.39–1.14)	0.10
Model 2	1.00	0.75 (0.45–1.26)	0.70 (0.40–1.21)	0.98 (0.96–1.01)	0.14
Model 3	1.00	0.88 (0.56–1.50)	0.84 (0.47–1.52)	0.98 (0.52–1.83)	0.85
Model 4	1.00	1.00 (0.58–1.74)	0.98 (0.54–1.79)	1.09 (0.56–2.11)	0.85
**Psychological distress**					
Crude	1.00	0.93 (0.66–1.31)	0.75 (0.57–1.09)	0.76 (0.52–1.12)	0.09
Model 1	1.00	1.04 (0.71–1.52)	0.74 (0.49–1.11)	0.67 (0.43–1.02)	0.03
Model 2	1.00	1.04 (0.70–1.54)	0.72 (0.47–1.10)	0.69 (0.45–1.07)	0.04
Model 3	1.00	1.12 (0.75–1.68)	0.80 (0.51–1.27)	0.83 (0.50–1.36)	0.27
Model 4	1.00	1.26 (0.80–1.84)	0.92 (0.58–1.47)	0.78 (0.46–1.33)	0.29

a *Model 1: Adjusted for age and energy intake*.

*Model 2: Further adjustment for physical activity, smoking, marital status, socioeconomic status, diabetes, and use of antidepressant medications and dietary supplements*.

*Model 3: Additional controlling for dietary intakes of fat, n-3 fatty acids, vitamin B group, and total antioxidants*.

*Model 4: Further adjusted for body mass index (BMI)*.

**Table 5:** Multivariable-adjusted odds ratios and 95% confidence intervals for depression, anxiety, and high psychological distress across quartiles of energy-adjusted dietary fiber intake in women (*n* = 1,959)^a^.

	**Quartiles of energy-adjusted dietary fiber intake**				
	**(*n* = 434) (<19 g/day)**	**(*n* = 465)(19–22.1 g/day)**	**(*n* = 514) (22.2–25.6 g/day)**	**(*n* = 546)(>25.6 g/day)**	***p*_**trend**_**
**Depression**					
Crude	1.00	0.65 (0.49–0.85)	0.70 (0.54–0.91)	0.59 (0.45–0.77)	0.001
Model 1	1.00	0.63 (0.47–0.83)	0.71 (0.54–0.93)	0.57 (0.43–0.75)	<0.001
Model 2	1.00	0.62 (0.46–0.82)	0.70 (0.53–0.93)	0.54 (0.41–0.72)	<0.001
Model 3	1.00	0.63 (0.47–0.85)	0.76 (0.56–1.02)	0.63 (0.46–0.88)	0.03
Model 4	1.00	0.61 (0.45–0.83)	0.74 (0.55–1.00)	0.63 (0.45–0.88)	0.03
**Anxiety**					
Crude	1.00	0.63 (0.45–0.88)	0.81 (0.59–1.10)	0.65 (0.47–0.89)	0.04
Model 1	1.00	0.61 (0.43–0.86)	0.80 (0.58–1.11)	0.61 (0.44–0.86)	0.03
Model 2	1.00	0.60 (0.42–0.86)	0.80 (0.57–1.12)	0.59 (0.42–0.83)	0.02
Model 3	1.00	0.57 (0.40–0.82)	0.77 (0.54–1.09)	0.56 (0.37–0.83)	0.03
Model 4	1.00	0.58 (0.40–0.84)	0.82 (0.57–1.19)	0.56 (0.37–0.85)	0.05
**Psychological distress**					
Crude	1.00	0.63 (0.48–0.84)	0.68 (0.52–0.89)	0.60 (0.57–0.89)	0.002
Model 1	1.00	0.64 (0.48–0.87)	0.75 (0.56–1.00)	0.63 (0.47–0.84)	0.009
Model 2	1.00	0.63 (0.47–0.86)	0.74 (0.55–1.00)	0.61 (0.46–0.82)	0.006
Model 3	1.00	0.63 (0.46–0.85)	0.75 (0.55–1.02)	0.64 (0.46–0.90)	0.04
Model 4	1.00	0.65 (0.47–0.89)	0.80(0.58–1.11)	0.70 (0.47–0.95)	0.09

a *Model 1: Adjusted for age and energy intake*.

*Model 2: Further adjustment for physical activity, smoking, marital status, socioeconomic status, diabetes, and use of antidepressant medications and dietary supplements*.

*Model 3: Additional controlling for dietary intakes of fat, n-3 fatty acids, vitamin B group, and total antioxidants*.

*Model 4: Further adjusted for body mass index (BMI)*.

Multivariable-adjusted ORs and 95% CIs for psychological disorders across different categories of dietary fiber consumption stratified by weight status (BMI ≥25 vs. <25 kg/m^2^) are provided in Tables 6, 7. Both in the crude model and adjusted model, no significant association between higher intake of dietary fiber and depression and anxiety was found in overweight or obese participants, while higher consumption of dietary fiber was related to reduced likelihood of having high psychological distress both in the crude model (OR: 0.68; 95% CI: 0.49, 0.94) and fully adjusted model (OR: 0.52; 95% CI: 0.34, 0.79).

**Table 6:** Multivariable-adjusted odds ratios and 95% confidence intervals for depression, anxiety, and high psychological distress across quartiles of energy-adjusted dietary fiber intake in overweight or obese participants [body mass index (BMI) ≥25 kg/m^2^] (*n* = 1,506)^a^.

	**Quartiles of energy-adjusted dietary fiber intake**				
	**(*n* = 351) (<19 g/day)**	**(*n* = 369)(19–22.1 g/day)**	**(*n* = 376) (22.2–25.6 g/day)**	**(n = 410)(>25.6 g/day)**	***p*_**trend**_**
**Depression**					
Crude	1.00	0.87 (0.64–1.19)	0.83 (0.60–1.13)	0.81 (0.59–1.10)	0.17
Model 1	1.00	0.87 (0.62–1.22)	0.81 (0.58–1.14)	0.71 (0.51–1.00)	0.05
Model 2	1.00	0.84 (0.59–1.20)	0.79 (0.56–1.12)	0.68 (0.48–0.96)	0.03
Model 3	1.00	0.93 (0.65–1.34)	0.93 (0.65–1.35)	0.87 (0.59–1.29)	0.52
**Anxiety**					
Crude	1.00	0.96 (0.65–1.42)	0.90 (0.61–1.34)	0.91 (0.62–1.34)	0.60
Model 1	1.00	0.80 (0.52–1.24)	0.84 (0.55–1.28)	0.82 (0.54–1.25)	0.44
Model 2	1.00	0.76 (0.49–1.19)	0.81 (0.53–1.26)	0.77 (0.50–1.18)	0.33
Model 3	1.00	0.78 (0.49–1.23)	0.88 (0.55–1.40)	0.84 (0.52–1.38)	0.67
**Psychological distress**					
Crude	1.00	0.71 (0.51–0.99)	0.68 (0.49–0.94)	0.68 (0.49–0.94)	0.02
Model 1	1.00	0.70 (0.49–1.00)	0.65 (0.46–0.93)	0.56 (0.39–0.79)	0.001
Model 2	1.00	0.68 (0.47–0.98)	0.61 (0.42–0.87)	0.51 (0.36–0.73)	<0.001
Model 3	1.00	0.67 (0.46–0.98)	0.61 (0.42–0.90)	0.52 (0.34–0.79)	0.002

a *Model 1: Adjusted for age, gender, and energy intake*.

*Model 2: Further adjustment for physical activity, smoking, marital status, socioeconomic status, diabetes, and use of antidepressant medications and dietary supplements*.

*Model 3: Additional controlling for dietary intakes of fat, n-3 fatty acids, vitamin B group, and total antioxidants*.

**Table 7:** Multivariable-adjusted odds ratios and 95% confidence intervals for depression, anxiety, and high psychological distress across quartiles of energy-adjusted dietary fiber intake in normal-weight participants [body mass index (BMI) <25 kg/m^2^] (*n* = 1,856)^a^.

	**Quartiles of energy-adjusted dietary fiber intake**				
	**(*n* = 489) (<19 g/day)**	**(*n* = 472)(19–22.1 g/day)**	**(*n* = 465) (22.2–25.6 g/day)**	**(*n* = 430)(>25.6 g/day)**	***p*_**trend**_**
**Depression**					
Crude	1.00	0.70 (0.53–0.92)	0.75 (0.57–0.98)	0.64 (0.48–0.85)	0.004
Model 1	1.00	0.68 (0.50–0.91)	0.70 (0.52–0.95)	0.58 (0.43–0.80)	0.001
Model 2	1.00	0.68 (0.50–0.92)	0.71 (0.52–0.96)	0.59 (0.43–0.81)	0.002
Model 3	1.00	0.70 (0.51–0.95)	0.78 (0.56–1.07)	0.74 (0.51–1.06)	0.14
**Anxiety**					
Crude	1.00	0.56 (0.39–0.80)	0.78 (0.80–1.09)	0.55 (0.38–0.81)	0.01
Model 1	1.00	0.54 (0.36–0.79)	0.73 (0.51–1.05)	0.48 (0.32–0.71)	0.002
Model 2	1.00	0.55 (0.37–0.82)	0.76 (0.52–1.10)	0.50 (0.33–0.75)	0.005
Model 3	1.00	0.55 (0.37–0.82)	0.75 (0.51–1.12)	0.50 (0.31–0.80)	0.02
**Psychological distress**					
Crude	1.00	0.79 (0.59–1.06)	0.82 (0.61–1.09)	0.74 (0.55–1.00)	0.07
Model 1	1.00	0.82 (0.60–1.13)	0.85 (0.62–1.16)	0.73 (0.53–1.00)	0.07
Model 2	1.00	0.82 (0.60–1.14)	0.86 (0.63–1.19)	0.74 (0.53–1.04)	0.11
Model 3	1.00	0.83 (0.60–1.16)	0.91 (0.65–1.29)	0.86 (0.59–1.26)	0.54

a *Model 1: Adjusted for age, gender, and energy intake*.

*Model 2: Further adjustment for physical activity, smoking, marital status, socioeconomic status, diabetes, and use of antidepressant medications and dietary supplements*.

*Model 3: Additional controlling for dietary intakes of fat, n-3 fatty acids, vitamin B group, and total antioxidants*.

Normal-weight individuals in the highest quartile of dietary fiber intake, compared with those in the lowest intake, had 36% lower risk to be depressed (OR: 0.64; 95% CI: 0.48, 0.85) and 45% lower risk to be anxious (OR: 0.55; 95% CI: 0.38, 0.81) in the crude model. After taking dietary intake into account, this relationship disappeared for depression. No relationship was found between dietary fiber consumption and high psychological distress in normal-weight participants.

## Discussion

We found inverse associations between dietary fiber intake and anxiety and high psychological disorder among Iranian adults. Higher consumption of dietary fiber was also associated with reduced odds of depression in women. These associations were independent of potential confounders, including lifestyle-related factors, dietary intakes, and anthropometric status of individuals. This research is one of the first epidemiological investigations that assessed the relationship between total dietary fiber consumption and mental disorders in a Middle Eastern population.

Psychological disorders are prevalent public health problems that affect quality of life and familial and social wellbeing and have an economic burden for individuals and societies (6, 7, 29). Our results showed that total dietary fiber intake might have a role in preventing anxiety, depression, and distress. Considering the pandemic prevalence of mental disorders, even a minimal advantage to reduce the prevalence of depression or anxiety would be substantial to the entire population.

We observed that more dietary fiber intake might link with reduced odds of anxiety and high psychological disorder. Moreover, total dietary fiber intake was associated with a lower risk of depression in Iranian women. Xu et al. (30) have conducted a cross-sectional study and reported that total fiber intake was associated with a reduced risk of depression in a non-linear manner, such that the risk of depression reached the lowest level among the population when total fiber was consumed ~21 g/day. The average intake of dietary fiber among most Americans is about 15 g/day, which is less than adequate intake (25 g/day for females and 38 g/day for males) (31). Thus, more intakes of the foods with high amounts of dietary fiber might help prevent psychological disorders (30). Other previous investigations have also confirmed these findings (18, 28, 32). More consumption of dietary fiber was linked to a lower risk of depression in postmenopausal women in Women's Health Initiative Observational Study (28). Consumption of dietary fiber has also reduced the risk of depressive symptoms in adolescent Korean girls (32). The same result was also obtained among 3,999 Chinese elders (18). In addition, Mihrshahi et al. (8) have shown that lower intake of fruit was associated with depression in middle-aged women. In this regard, a cross-sectional analysis among adults showed that the linkage between mental health and the GI microbiota could be affected by both participant's sex and dietary fiber intake (33). Randomized controlled trials have also supported that consumption of prebiotic fibers could improve mood and behaviors (34–36). Supplementation with 5.0 g/day fructo-oligosaccharides and 7.0 g/day trans-galacto-oligosaccharides could alter the gastrointestinal microbiota and improve mood (34, 36).

In contrast, some other investigations reported non-significant inverse associations between fiber intake and depressive symptoms. In older Australian adults, no significant association was found between intakes of fiber from fruit, vegetable, breads, or cereals with depression (37). Similar results were obtained in elderly Japanese males and females (9). It is worth noting that both of these mentioned investigations were carried out among participants with the age of 65 years or more who were at risk of mental disorder and had different lifestyles and poor nutrition. Also, the effects of potential confounders including dietary intakes, sleep, and exercise were not taken into account in these researches (9). Furukawa Nutrition and Health Study suggested that intake of fiber from vegetables and fruits was inversely related to depression in Japanese adults, while no association was found between total, soluble, or insoluble dietary fiber or cereal fiber and depression. The inconsistent findings of previous investigations might be partly related to the differences in age, gender, ethnicity, or race and different dietary fiber intake levels of the study participants (6).

Overall, fiber represents a very broad class of structurally complex compounds. In the current study, we examined the relationship between total dietary fiber intake and psychological disorders; unfortunately, we could not differentiate the type of consumed dietary fiber due to the lack of sufficient information for some food components in the Iranian nutrient database. Our applied method to assess dietary fiber intake might be common in the field but from a nutritional point of view could face some limitations. Fiber provides direct physical benefits, including increased fecal bulking and laxation. Insoluble dietary fiber or fiber sources such as inulin that are used as prebiotics have also general health-promoting effects. However, another feature of dietary fiber—a nutrient category that includes a broad array of non-starch polysaccharides (such as arabinoxylans and β-glucans) and other fiber sources that are not digestible by human enzymes—has also drawn it into the spotlight: it provides an important substrate to the community of microbes (microbiota) that inhabits the distal gut (38). So, it could increase the viscosity of digesta and promote the growth of pathogens or even dysbiosis, which in turn might enhance the production of detrimental microbial metabolites such as lipopolysaccharides or biogenic amines. This aspect could also explain previous contradictory findings. Stratified analysis of fiber sources in future prospective investigations has been suggested to shed a light on these associations.

In the current study, a significant relationship was found between dietary fiber intake and depressive symptoms in females, but not in males. One reason could be healthier dietary intakes and more fiber intake in women than men (22.96 vs. 21.86 g/day, *p* < 0.001). The higher prevalence of depression (35.1 vs. 22.9%, *p* < 0.001) among women could be another reason for this finding. Also, women are more comfortable than men in expressing their problems and illnesses and less likely to hide their psychological disorders.

The evidence that explains mechanisms of the relation of dietary fiber and mental health is not completely understood, but several possibilities have been suggested. Gut microbiota may play a positive role in neurotransmitter metabolism such as serotonin synthesis (39). Dietary fiber consumption could improve intestinal flora composition (40). These flora could communicate with the central nervous system (41). So, dietary fiber could have a beneficial effect on mood through gastrointestinal microbiota, but epidemiologic investigations in the field of dietary fiber intake and mood relation in free-living populations are scarce. A bidirectional link between the gut microbiota and the brain, known as gut–brain axis, is a pathway that is mediated by immune, humoral, and neural systems (39). Furthermore, fermentation of dietary fiber produced short-chain fatty acids that improve inflammatory responses (41) as a basic pathway in depressive symptoms (42). Intake of dietary fiber might also control postprandial hyperglycemia, decrease oxidative stress, and inhibit the inflammatory processes in this way (43–45).

The strengths of the current study were its large sample size with a relatively high response rate and the wide range of age groups. The effects of total energy intake and other confounders were also considered in the analysis. We used validated tools to assess the exposure and outcomes of interest. However, the current study had several limitations. It has a cross-sectional design that would not allow us to reach a causal relationship, although the appropriate analysis of cross-sectional data could be an initial step to better understand the diet–disease relationship. Furthermore, the possibility impact of unmeasured confounders or residual confounding cannot be excluded, although we made adjustments for several potential confounders. HADS was a subjective screening instrument for depressive symptoms, not an objective tool to define clinical depression; thus, misclassification bias might occur. So, no objective measures of psychological symptoms as well as dietary intakes were used. Some sort of recall bias was also inevitable due to assessment of dietary intakes in the preceding year through an FFQ. In addition, we included the lean participants in the category of normal-weight subjects due to the low number of these individuals in our sample (*n* = 114). Depression and anxiety were more prevalent among lean subjects than normal-weight individuals (41.2 vs. 30% for depression and 16.7 vs. 15.0% for anxiety). So, some sort of bias might occur because of this inclusion; however, it would be negligible and such error would move the estimates toward the null. Our study population involved medical university non-academic staff, including crews, employees, and managers. To reduce the conflict of interest in the research, some University teaching hospitals and research centers were not included. So, <8% of participants had a master's and above educational level. The wide range of SES of the study population was also representative of the general Iranian population; however, generalizability of the findings to other populations should be made with caution. Finally, this investigation did not take the source of dietary fiber into account, which is proposed to be considered in future researches.

In conclusion, significant inverse associations between dietary fiber intake with anxiety and high psychological distress were found in Iranian adults in this cross-sectional study. More consumption of dietary fiber was also related to reduced odds of depression in women. More investigations with a prospective nature are needed to affirm these findings.

## Data Availability Statement

The data analyzed in this study is subject to the following licenses/restrictions: Dataset can be available on request from supervisor of the project (AE). Requests to access these datasets should be directed to a.esmaillzadeh@gmail.com.

## Ethics Statement

The study was ethically approved by the Research Council of Tehran University of Medical Sciences, Tehran, Iran (TUMS). The patients/participants provided their written informed consent to participate in this study.

## Author Contributions

FS, NS, PS, AK, MH-A, HA, AE, and PA designed the research, conducted the study, analyzed the data, wrote the manuscript, and had the responsibility for the final content. All authors read and approved the final manuscript.

## Conflict of Interest

The authors declare that the research was conducted in the absence of any commercial or financial relationships that could be construed as a potential conflict of interest.

## Abbreviations

FFQ: food frequency questionnaire
SEPAHAN: Study on the Epidemiology of Psychological-Alimentary Health and Nutrition
GHQ: General Health Questionnaire
HADS: Hospital Anxiety and Depression Scale
IUMS: Isfahan University of Medical Sciences
OR: odds ratio
95% CI: 95% confidence interval
SES: socioeconomic status
GPPAQ: General Practice Physical Activity Questionnaire
ANOVA: analysis of variance
ANCOVA: analysis of covariance.
